# Severest crisis overlooked—Worst disruption of terrestrial environments postdates the Permian–Triassic mass extinction

**DOI:** 10.1038/srep28372

**Published:** 2016-06-24

**Authors:** Peter A. Hochuli, Anna Sanson-Barrera, Elke Schneebeli-Hermann, Hugo Bucher

**Affiliations:** 1 Institute and Museum of Palaeontology, University of Zurich, Karl Schmid-Str. 4, CH-8006 Zurich, Switzerland

## Abstract

Generally Early Triassic floras are believed to be depauperate, suffering from protracted recovery following the Permian–Triassic extinction event. Here we present palynological data of an expanded East Greenland section documenting recovered floras in the basal Triassic (Griesbachian) and a subsequent fundamental floral turnover, postdating the Permian–Triassic boundary extinction by about 500 kyrs. This event is marked by a swap in dominating floral elements, changing from gymnosperm pollen-dominated associations in the Griesbachian to lycopsid spore-dominated assemblages in the Dienerian. This turnover coincides with an extreme δ^13^C_org_ negative shift revealing a severe environmental crisis, probably induced by volcanic outbursts of the Siberian Traps, accompanied by a climatic turnover, changing from cool and dry in the Griesbachian to hot and humid in the Dienerian. Estimates of sedimentation rates suggest that this environmental alteration took place within some 1000 years. Similar, coeval changes documented on the North Indian Margin (Pakistan) and the Bowen Basin (Australia) indicate the global extent of this crisis. Our results evidence the first profound disruption of the recovery of terrestrial environments about 500kyrs after the Permian–Triassic extinction event. It was followed by another crisis, about 1myrs later thus, the Early Triassic can be characterised as a time of successive environmental crises.

Scientists as well as the broad public become more and more aware of modern (manmade) destruction of terrestrial environments caused not only by the obvious effects of air pollution or clearing rain forests but also by the more subtle and long-term climatic change^1^,^2^. Deforestation is associated with the loss of biodiversity, increased greenhouse gas emissions, disrupted water cycles, increased soil erosion, and disrupted lebensraum^3^,^4^,^5^,^6^,^7^. These effects are also regularly discussed as consequences of the events leading to the Phanerozoic mass extinctions^8^. In fact there is increasing evidence that we are currently witnessing the sixth–manmade-mass extinction, associated with the destruction of natural environments and climatic change^9^. Thus, the hypothesis published a few years ago that the Permian–Triassic mass extinction (PTME), the biggest extinction event in Earth history (ca. 251myrs ago), was associated with an essentially global deforestation^10^,^11^ was easily accepted by the scientific community. This led to the widely approved concept that the destruction of the gymnosperm forests induced a pioneer vegetation of herbaceous lycopsids that subsequently dominated the terrestrial environments during the entire Early Triassic. Furthermore, the climate of the Early Triassic was interpreted as homogeneously warm and semi-arid^12^ or hot and arid^13^,^14^. These ideas concurred with the commonly hawked hypothesis that Early Triassic biotas reflect a protracted recovery after the PTME. Recent studies, however, show a more complex picture of the development of the biosphere and climate during this time, providing evidence for significant climatic changes^15^ as well as for profound faunal and floral turnovers^16^,^17^,^18^,^19^,^20^,^21^,^22^,^23^,^24^,^25^. New evidence suggest that not all marine organisms were similarly affected by the PTME. Most linages of conodonts and numerous radiolarians survived this event, but went extinct around the Griesbachian–Dienerian boundary while typical Mesozoic conodont and radiolarian assemblages appeared after this event^17^,^18^. Other Griesbachian records have reported “incredibly diverse benthic fauna”^19^, and “unexpectedly diverse and complex ichnofauna”^20^, which reveal at least locally sound living conditions for benthic ecosystems. Hence, these findings suggest that during the Griesbachian environmental conditions were less hostile than generally assumed and that an important extinction event affecting several groups occurred around the Griesbachian–Dienerian boundary^17^,^18^,^20^,^23^. Palynological studies of expanded sections with well-preserved pollen and spores have provided insight into the dynamics of the development of plant associations during these critical intervals. New results show that the PTME lead to short term changes in the plant community abundance structure, expressed in the palynological records by a “spore spike”^10^,^24^,^26^. Thus, the immediate reaction of the plant communities on the environmental catastrophe of the PTME was similar to those known from the subsequent major cataclysms, such as at the Triassic–Jurassic boundary (TJB) or the Cretaceous-Paleogene events (KPE), which are also characterized by short-lived super-abundance of pteridophyte spores^27^,^28^,^29^,^30^. These “spore spikes” systematically concur with negative δ^13^C isotope shifts. Thus, they obviously reflect reaction of the vegetation to environmental cataclysms, such as massive volcanic outbursts related to the PTME or the TJB^28^,^31^,^32^ or to the asteroid impact at the KPB^33^. This shows that different causes of disruption of terrestrial ecosystems lead to a similar reactions of the vegetation, even concerning various groups of pteridophytes–lycopsids at the PTME versus ferns at the TJB and the KPB. In fact in modern disturbed ecosystems ferns and fern allies play an important role in ecosystem restoration due to their tolerance to grow in acidic water-logged conditions or their ability to exploit nutrient-poor substrates–conditions that are associated with volcanic outbursts^29^,^34^,^35^. After the clearance of primary forests these opportunistic plants are known to take over and give subsequently way to the succeeding forests^34^,^36^. High resolution palynological data including the PTME indicate that gymnosperms recovered probably within a few kyrs to become again the dominating floral element^24^. Some typical Permian floral elements (e.g., *Vittatina*) gradually disappeared during this phase of recovery, although without an obvious loss in biodiversity or extinction of major plant groups^24^,^37^. Instead the detailed records from the Finnmark Platform^24^,^37^ show a distinct diversification of both gymnosperms and pteridophytes, which results in increased diversities after the PTME. New radiometric datings of the Permian–Triassic succession of the Bowen Basin^38^ shed a new light on the impact of PTME on plants. According to this new calibration the *Playfordiaspora crenulata*–and the *Protohaploxypinus microcorpus* zones, formerly attributed to the Late Permian, are now dated as Induan. Consequently, the floral turnover between the two zones described by Foster^39^ reflects now a mid-Induan event. The palynological assemblages of the *P. crenulata* zone are characterised by high abundance and diversity of taeniate bisaccate pollen changing at the boundary to the *P. microcorpus* zone to a dominance of lycopsid spores. Above all this event is marked by the disappearance of the *Glossopteris* flora^39^.

The Griesbachian record from East Greenland and the coeval section in the Trøndelag area of mid-Norway show well diversified spore-pollen assemblages, with clear dominance of gymnosperms^40^. This evidence is in obvious contrast with the previous general conception of depauperate Early Triassic sporomorph assemblages that are assumed to be characterized by overall dominance of lycopsid spores^12^. The present study of the expanded Kap Stosch section (Hold with Hope, East Greenland, 73°60′N/21°12′W–74°04′N/21°43′W) reveals that the profound and sustained floral change leading to the dominance of lycopsids happened ca. 500 kyrs after the PTME^21^,^41^,^42^,^43^ around the Griesbachian–Dienerian boundary (GDB). Here the gymnosperm pollen dominated floral associations of the basal Triassic (Griesbachian) changed to lycopsid spore-dominated assemblages. Evidence from other areas (e.g., Barents Sea, Pakistan, Tibet) shows that lycopsid subsequently dominated the flora up into the middle Smithian, for about 1myrs where other floral changes of global extent led to a renewed and short lived dominance of gymnosperms in the late Smithian^22^,^23^,^44^. Late Early Triassic (Spathian) floras are characterised by mixed assemblages showing a general decline of lycopsid spores associated with renewed diversification of terrestrial floras towards the Middle Triassic (e.g., 23, 45, 46).

## Results

**Figure 1:**
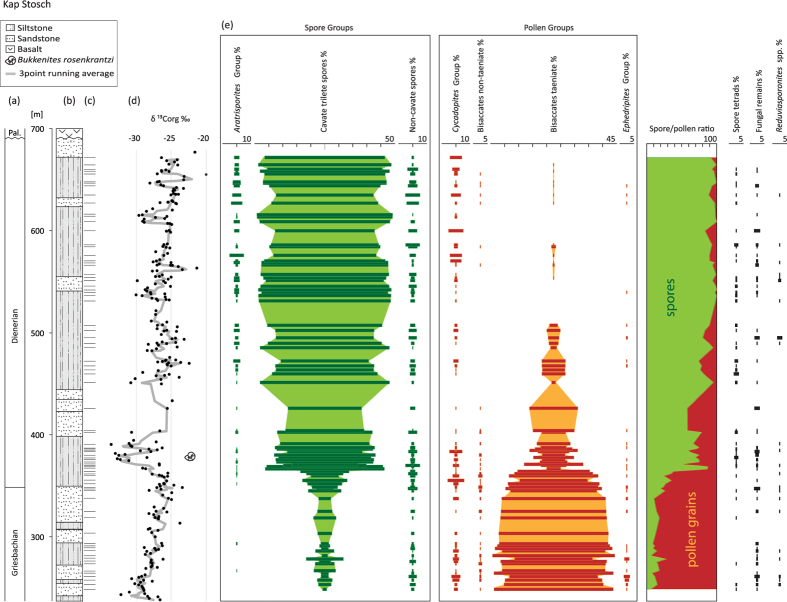
Overview on Griesbachian–Dienerian floras with generalized C-isotope curve. (**a**) Age (**b**) lithology (**c**) sample levels (**d**) bulk organic carbon isotopes^46^ (**e**) palynological data including relative abundances of groups of spore and pollen grains, spore/pollen ratios as well as spore tetrads, fungal remains and *Reduviasporonites* spp.

Here the general bulk palynological record with over 80 samples covering the upper part of the Griesbachian and the Dienerian from Kap Stosch is presented. The studied section is part of the Late Permian–Early Triassic Greenland-Norway rift basin. Deltaic sediments of the Wordie Creek Formation with changing marine influence were deposited during the latest Permian and the Induan (Griesbachian and Dienerian). The Griesbachian part of the formation comprises about 270m of section of which the upper 110 metres are included in the present study (Fig. 1). Age control is provided by a few horizons with ammonoids and bulk organic carbon isotope chemostratigraphy^47^,^48^. The Griesbachian–Dienerian boundary (GDB) is placed at the onset of a negative δ^13^C_org_ shift with the ammonoid *Bukkenites rosenkrantzi* occurring just above this level. Diverging from the interpretation of Bjerager *et al*.^45^ *B. rosenkrantzi* is considered here to be of Dienerian age because the worldwide oldest occurrences of proptychitids to which this species belongs are of Dienerian age or younger^21^.

For the present study we differentiated seven main floral elements, which are mentioned below together with the essential changes in their distribution (Fig. 1). The first group–***Aratrisporites***-is generally associated with the macrofossils *Annalepis*, a probably herbaceous lycopsid of isoetalean affinity^47^. This group, being very rare in the Griesbachian, becomes a significant element in the upper part of the section. **Cavate trilete spores**, comprising essentially the genera *Densoisporites, Kraeuselisporites*, and *Lundbladispora* can be attributed to lycopsids, probably also to the Isoetales^47^. They show average abundances of 10–20% in the Griesbachian; increasing around the GDB (at ca. 350 m) to about 60%, to become the overwhelmingly dominant group with up to 90% of the assemblages in the uppermost part of the section (from ca. 450 m onward). **Non-cavate spores** comprise a variety of smooth and ornamented spores, which represent various pteridophytes (lycopsids and ferns) but also bryophytes. This group shows no significant abundance change within the record. Pollen grains summarized under ***Cycadopites*** **group** comprise a great variety of possible gymnosperm parent plants (e.g., Bennettiales, Ginkgoales, Cycadales, and Peltaspermales)^47^. They are relatively common in the lower part of the succession and up to 10% in some samples of the GDB interval. They represent essentially the only group of gymnosperms (up to 10%) in the uppermost part of the studied section. **Non-taeniate bisaccate pollen grains** include conifers but also some groups of pteridosperms. This group is rare in the upper part of the Griesbachian and almost disappears in the Dienerian. **Taeniate bisaccate pollen grains**, attributed to pteridosperms, represent the dominant group in the lower part of the record with abundances up to 90%. Around the GDB their abundance decreases gradually over a short interval from around 60% to about 20%, and they become rare (<5%) in the uppermost part of the section. The ***Ephedripites*** **group**, representing Gnetales^47^, is regularly observed in the lowermost part of the record with abundances up to 5% and occurs only sporadically in the upper part of the section. The **spore/pollen ratio** summarizes the essential changes in the plant assemblages. This ratio is regarded as a rough indicator for water availability to the plant communities^24^,^48^ Because they have played a significant role in the interpretation of the environmental disaster of the PTME^11^,^49^ the quantitative distribution of **spore tetrads** (comprising essentially *Densoisporites* spp.), **fungal remains**, and ***Reduviasporonites*** **spp.** are also included. In the present record their abundances show no significant trend. Generally, the trends in the distribution of all the above mentioned groups are unrelated to changes in the depositional environment^46^.

In the Kap Stosch area the Induan (Griesbachian and Dienerian) interval comprises about 600 m of section^46^. Thus, accepting the roughly estimated duration of the Induan of about 1.0 myr^41^,^42^,^43^ would result in an average sedimentation rate of ca. 0.6 m/kyr in the studied section. The estimated duration of the Dienerian at 416 kyrs by Ware *et al*.^21^ would suggest a sedimentation rate of 0.79 m/kyr for the 328 m of Dienerian section, provided that the entire substage is represented. The described mid-Induan floral turnover takes place over an interval of a ca. 4 metres at the onset of the prominent δ^13^C_org_ shift close to the GDB, thus we conclude that the changes occurred over a very short interval in the order of a few kyrs.

## Discussion

Compared to the marine fauna relatively little is known about the impacts of the PTME and following events on the terrestrial realm. The record of Early Triassic plant macrofossils is extremely poor and mostly uncalibrated; however, palynological records adequately reflect floral changes during mass extinctions and the subsequent recovery phases^22^,^23^,^25^. For the PTME some spectacular scenarios have been inferred from relatively few records, e.g., total extinction^50^ totally devastated environments unsuited for plants^12^,^49^,^51^,^52^, plant mutagenesis due to ozone layer destruction^10^, and collapse of terrestrial ecosystems related to a fungal event^49^,^52^. Evidence for the latter event is still questionable, mainly because of the ambiguous biological attribution of *Reduviasporonites*, the key witness of this event, assigned by some authors to fungi^53^ and by others-with more convincing evidence-to algae^54^,^55^. However, high abundances of *Reduviasporonites*, as those interpreted as “fungal event”, have not been observed in any expanded section. *Reduviasporonites* occurs regularly but not abundantly throughout these records^24^,^37^,^39^.

Another effect of the PTME has been inferred from high numbers of spore tetrads in sediments comprising this event. It has been proposed that stratospheric ozone layer depletion and subsequent increased UV-B radiation caused genetic damage (mutagenesis) on lycopsids^11^. According to these authors the effect of this event prevented the spore tetrads of some lycopsids to separate during maturation. In fact peak abundances of tetrads coincide with the above mentioned “spore spike”. On the other hand, the tendency to shed the spores in tetrads could also be inherent to the lycopsid groups in question, as it is known for other fossil plants, such as the conifer pollen *Triadispora* or *Classopollis*. Other authors relate the high abundance of tetrads to depositional environments adjacent to the plant’s habitat^56^. Thus, the reasons for these occurrences remain ambiguous. In all expanded palynological records (Kap Stosch, Trøndelag and Finnmark platform) spore tetrads are regularly observed throughout the Griesbachian and the Dienerian. In the Kap Stosch record levels with increased numbers seem to be randomly distributed and are certainly unrelated to the GDB event (Fig. 1).

Due to the lack of adequate sections, preservational bias, or lack of stratigraphic calibration basal Early Triassic terrestrial successions are relatively poorly known. Numerous palynological studies of the Late Permian contain relatively rich assemblages whereas the overlying Triassic sections appear impoverished or consist of a few samples only^12^,^55^,^57^,^58^,^59^,^60^ and therefore emphasise the impression of an impoverished Early Triassic flora. A few well calibrated quantitative palynological records exist for the basal Triassic of the Barents Sea area as well as for the classical Early Triassic sections in the Salt Range and the Surghar Range in Pakistan, and based on recent radiometric age datings, for the Bowen Basin (eastern Australia)^23^,^24^,^25^,^37^,^39^,^40^. Calibration of the so far poorly constrained Australian Permian–Triassic palynostratigraphic succession shows that the most important floral turnover, associated with the extinction of the glossopterids, happened not at the Permian–Triassic boundary but within the Induan. Similar to the changes observed in E-Greenland this turnover is expressed by a strong reduction of the abundance and diversity of the pteridosperms (taeniate bisaccate pollen) and by a corresponding increase in lycopsids^38^,^39^.

The present record from East Greenland together with other high resolution terrestrial datasets challenges some of the running hypothesis associated with the PTME and its consequences for the Early Triassic flora. In contrast to the above mentioned spectacular extinction scenarios, the data from expanded latest Permian–earliest Triassic interval from the Southern Barents Sea (Finnmark platform) shows a distinct “spore spike” in reaction to the PTME^37^, i.e. reflecting not an extinction event, but a shift in the vegetation’s abundance structure. This event is also documented in a coeval section in East Greenland^10^,^26^. The data from the Finnmark platform^24^ and from E-Greenland^10^ suggest that this spore spike was of short duration–in the order of 10 kyrs. The succession following this “spore spike”, well documented in the Finnmark section, suggests a return to conditions similar to pre-event status, although with a gradual loss of some typical Late Permian elements during the Griesbachian^24^,^37^,^40^. The detailed study of this section shows the disappearance of several species–namely of the *Vittatina* group-near the PTB associated with numerous taxa appearing near this level^7^,^61^. The Griesbachian assemblages from Kap Stosch illustrate a rich and diverse flora subsequent to the PTME, similar to coeval records previously documented from the Finnmark and the Trøndelag platforms^40^. For eastern Australia Foster^39^ described the difference between the palynozone “Upper Stage 5”, now attributed to the Late Permian, and the basal Induan *P. crenulata* zone as gradual, marked only by the first occurrence of a few marker species. Thus the evidence for a sudden plant extinction related to the PTME becomes more and more scanty.

The presented floral record provides a so far unique insight on environmental changes during the aftermath of the PTME. Pronounced dominance of pteridosperms can be inferred from the lower part of the Induan records from the Northern hemisphere (Kap Stosch area, Finnmark and Trøndelag platforms) as well as from eastern Australia^39^. In the Kap Stosch section we can document a take-over of lycopsid spores of isoetalean affinity around the calibrated GDB. This turn-over is followed by a further decline of the gymnosperms during the Dienerian, ending in the top part of the section with an overall dominance of the lycopsids. The first decisive floral change coincides with the onset of a marked negative δ^13^C_org_ isotopic shift^46^. A subsequent change to the total dominance of spores in the middle part of the Dienerian concurs with the onset of another, although minor, negative δ^13^C_org_ shift (Fig. 1).

Comparable quantitative palynological records of Griesbachian and especially of Dienerian age from other areas are extremely rare. However, Schneebeli-Hermann *et al*.^25^ documented a coeval succession from the Salt Range (Pakistan). Although this section is strongly condensed (4m of section), it shows for the Griesbachian a strong dominance of gymnosperms-including pollen grains of glossopteridalean affinity, traditionally considered Permian markers, together with Triassic floral elements (e.g., Corystospermales, *Dicroidium* spp.)-followed by a gradual change to spore dominance in the Dienerian. In the Bowen Basin the significant decrease in the diversity and abundance of gymnosperms and the corresponding increase of lycopsids, coinciding with the disappearance of glossopterids, falls within the Induan, without further precision^38^,^39^. Detailed faunal data from the Salt Range sections reveals a low diversity ammonoid fauna at the base of the Dienerian, followed by a further diversity decrease in the middle Dienerian, and by a slight increase in the late Dienerian and finally by a significant diversification in the early Smithian^21^. Supposing that these ammonoid faunas reflect the general environmental quality, the middle Dienerian faunal assemblages would suggest most hostile conditions. The almost total demise of the gymnosperms in the Kap Stosch section might coincide with this faunal crisis and confirm a severe and prolonged global environmental crisis during the Dienerian.

**Figure 2:**
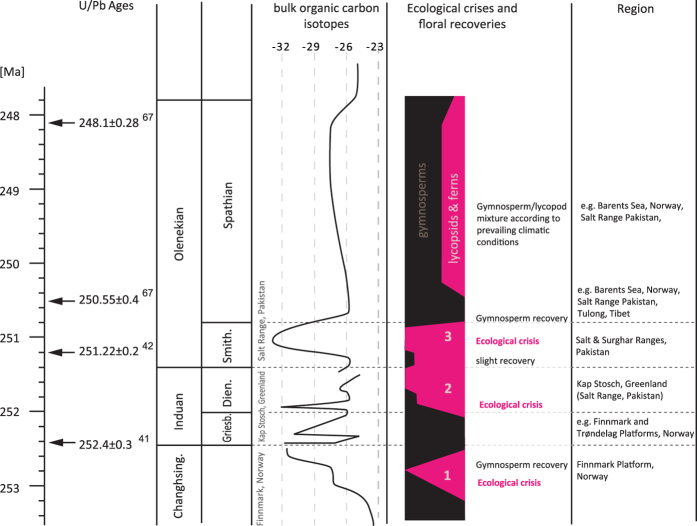
Summary of Early Triassic floral events. Radiometric ages^41^,^42^,^67^ together with a simplified bulk organic carbon isotope curve^24^,^48^,^50^ are shown in relation to floral events documented in the Boreal Realm^22^,^24^,^26^,^45^ and on the North Indian margin^23^,^25^,^44^.

Generally climatic changes are the primary trigger for changes in vegetation patterns. Fast reactions of the plant communities-within time spans of 100 yrs-are reliably recorded in changes in pollen assemblages (e.g., Early Holocene afforestation in the Alps^62^). In order to understand terrestrial environmental changes in the Early Triassic indicated by pronounced negative δ^13^C_org_ shifts and the coeval floral turnovers we try to infer the corresponding climatic conditions. Proliferation of spores is generally associated with more humid conditions, based on the fact that pteridophytes need liquid water at least during part of their life cycle. Thus spore dominated assemblages are considered to reflect relatively humid conditions. Temperature values are much more delicate to infer from plant records. Recent data of δ^18^O measurements of pristine biogenic apatite of conodonts reveal considerable changes in temperatures near the PTME and during the Early Triassic^15^. These authors inferred relatively cool temperatures for the Griesbachian and a temperature increase for the Dienerian. The δ^18^O record of the Early Triassic follows the trends in the carbon cycle reflected in the δ^13^C curve. The marked shifts in these records, reflecting severe environmental disturbances, are accompanied by major changes in plant assemblages (Fig. 2). For the Salt Range sections the increased spore ratios near the GDB and in the middle Smithian can be directly linked to negative shifts of δ^13^C_org_ and to lower δ^18^O^15^,^23^. These trends are interpreted to correspond to higher pCO_2_ and to higher temperatures that apparently induced at least seasonally increased humidity^15^. In contrast, the gymnosperms dominated assemblages of the late Smithian/early Spathian are associated with relatively positive δ^13^C_org_ values and δ^18^O values reflecting relatively cool temperatures^15^. Applying this relationship to the Induan succession of Kap Stosch, we suggest that the gymnosperm pollen dominated Griesbachian assemblages reflect relatively cool and dry conditions, which rapidly changed to hot and humid at the GDB.

Similar to the GDB event the onset of the δ^13^C negative shift leading to the minimum near the PTB, coincides with a sudden increase of spores^24^. Similar relationships have been observed in the middle Smithian^23^; near the TJB^28^,^32^,^63^ and near the KPB^64^ where spore maxima are also associated with negative δ^13^C shifts. These changes reflect severe environmental and climatic changes, which in the case of the Early Triassic are probably associated with effects of successive volcanic outbursts related to the Siberian Large Igneous Province. Within the studied interval the dominance of lycopsids got stepwise accentuated, with increasing percentages of herbaceous forms, which become regular elements of the vegetation during the Dienerian. In contrast to the PTME, where the vegetation is interpreted to have recovered within a short time, the dominance of the lycopsids, appearing at the GDB, lasted–with a slight recovery in the early Smithian-for several 100 kyrs, up to the late Smithian^23^. Similar changes documented for the mid Induan of the North Indian Margin and eastern Australia suggest a global extent of this event. Thus, the GDB event, reflected in the floral turn-over associated with the extinction of the glossopterids, the pronounced negative δ^13^C_org_ shift, and the extinction in some marine groups (conodonts and radiolarians) is interpreted to represent another severe environmental disruption in a series of events such as at the PTB-, the mid-Smithian-, the TJB or the KPB. Recent progress in the technique of radiometric dating enables us to calibrate changes in continental sections and to put these deep-time environmental cataclysms within precise temporal frameworks and make them comparable to rapid changes that happened during the last 100kyrs. The floral turnovers related to the PTME and to the GDB event are thought to have happened within a few kyrs; thus, they become realistic models for future changes.

## Methods

Sampling for palynology essentially focussed on fine grained, dark siltstones and mudstones; gaps in the record correspond to coarse clastic intervals. For the studied section 94 samples have been prepared following standard palynological preparation technique^23^ 80 samples contain well preserved sporomorphs. For each sample a minimum of 250 sporomorphs has been counted. The detailed description of the section, palynofacies analysis, isotope measurements and palaeoenvironmental interpretations has been previously published by Sanson-Barrera *et al*.^48^.

## Additional Information

**How to cite this article**: Hochuli, P. A. *et al*. Severest crisis overlooked—Worst disruption of terrestrial environments postdates the Permian–Triassic mass extinction. *Sci. Rep*. **6**, 28372; doi: 10.1038/srep28372 (2016).
